# Efficiency of the Hydroponic System as an Approach to Confirm the Solubilization of CaHPO_4_ by Microbial Strains Using *Glycine max* as a Model

**DOI:** 10.3389/fpls.2021.759463

**Published:** 2021-10-29

**Authors:** Mateus Neri Oliveira Reis, Layara Alexandre Bessa, Andressa Pereira de Jesus, Fabiano Guimarães Silva, Marialva Alvarenga Moreira, Luciana Cristina Vitorino

**Affiliations:** ^1^Laboratory of Agricultural Microbiology, Instituto Federal Goiano – Rio Verde Campus, Highway Sul Goiana, Rio Verde, Brazil; ^2^Laboratory of Plant Mineral Nutrition and CEAGRE – Exponential Agriculture Center of Excellence, Instituto Federal Goiano, Rio Verde, Brazil; ^3^Empresa de Pesquisa Agropecuária de Minas Gerais (EPAMIG), Santa Rita Experimental Field, Prudente de Morais, Brazil

**Keywords:** phosphate solubilizing microorganisms, PSM, bio-inputs, plant nutrition, plant growth promotion

## Abstract

The sustainable development of agriculture can be stimulated by the great market availability of bio-inputs, including phosphate-solubilizing microbial strains. However, these strains are currently selected using imprecise and questionable solubilization methodologies in solid or liquid media. We hypothesized that the hydroponic system could be a more efficient methodology for selecting phosphate-solubilizing strains as plant growth promoters. This methodology was tested using the plant *Glycine max* as a model. The growth-promoting potential of the strains was compared with that of the Biomaphos® commercial microbial mixture. The obtained calcium phosphate (CaHPO_4_) solubilization results using the hydroponic system were inconsistent with those observed in solid and liquid media. However, the tests in liquid medium demonstrated poor performances of *Codinaeopsis* sp. (328EF) and *Hamigera insecticola* (33EF) in reducing pH and solubilizing CaHPO_4_, which corroborates with the effects of biotic stress observed in *G. max* plants inoculated with these strains. Nevertheless, the hydroponic system allowed the characterization of *Paenibacillus alvei* (PA12), which is also efficient in solubilization in a liquid medium. The bacterium *Lysinibacillus fusiformis* (PA26) was the most effective in CaHPO_4_ solubilization owing to the higher phosphorus (P) absorption, growth promotion, and physiological performance observed in plants inoculated with this bacterium. The hydroponic method proved to be superior in selecting solubilizing strains, allowing the assessment of multiple patterns, such as nutritional level, growth, photosynthetic performance, and anatomical variation in plants, and even the detection of biotic stress responses to inoculation, obtaining strains with higher growth promotion potential than Biomaphos®. This study proposed a new approach to confirm the solubilizing activity of microorganisms previously selected *in vitro* and potentially intended for the bio-input market that are useful in P availability for important crops, such as soybeans.

## Introduction

The world population is estimated to reach ~9.735 billion people by 2050 (United Nations, 2019). Associated with the pressure for agriculture to meet the needs of human development, it is necessary to implement sustainable agricultural practices that increase productivity integrated with conservation measures (Taveira et al., 2019). The current productivity of important crops such as soybean is affected by phosphorus (P) deficiency in acidic soils since P plays a key role in the symbiotic capacity of nitrogen (N_2_) fixation (Sulieman and Tran, 2017; Wang et al., 2020; Jaiswal et al., 2021). Current agricultural practices use fertilizers to add P to the soil, however, the use of phosphate fertilizers is expensive and unsustainable (Situmorang et al., 2015). Nonetheless, these fertilizers are easily precipitated with aluminum (Al), iron (Fe), and calcium (Ca), forming low-solubility complexes that are not used by plants (Penn and Camberato, 2019). In this context, the dissemination of more economical and ecologically appropriate technologies to improve P availability in soil has become urgent.

Currently, the use of phosphate-solubilizing microbes (PSMs) has been shown as a promising method since they play a key role in P dynamics in soil and the subsequent availability of this element to plants (Islam and Hossain, 2012; Kafle et al., 2019). PSMs fractionate insoluble P forms into soluble forms through various biological mechanisms, including the production of organic acids and extracellular enzymes, which convert insoluble forms of P into forms available for plant absorption (Hanif et al., 2015; Li et al., 2015; Baliah et al., 2016; Gurikar et al., 2016; Doilom et al., 2020; Sarr et al., 2020; Zúñiga-Silgado et al., 2020). The organic acids produced include glycolic, 2-ketogluconic, acetic, citric, propionic, succinic, tartaric, formic, fumaric, lactic, malic, butyric, gluconic, valeric, oxalic, and citric acids (Hwangbo et al., 2003; Chen et al., 2006; Patel et al., 2008; Scervino et al., 2010; Zhu et al., 2012; Jog et al., 2014; Mehta et al., 2015; Yadav et al., 2015). However, despite their importance, few PSM strains are currently available in the global market for inoculants. This is partly due to faulty strain selection mechanisms, which often present a high potential for *in vitro* systems but a reduced potential for field systems.

Phosphate-solubilizing microbes strains are usually selected through screening in solid media containing phosphate sources, such as calcium phosphate (CaHPO_4_), Ca_3_(PO_4_)_2_, aluminum phosphate (AlPO_4_), or iron phosphate (FePO_4_). In these media, halo measurements determine the efficiency of the isolate in solubilizing the insoluble phosphate source. However, this qualitative method is considered inefficient for selection, as many isolates that do not produce any visible halo on solid media can solubilize various types of insoluble inorganic phosphates in liquid media (e.g., Nautiyal, 1999; Bashan et al., 2013; Salcedo et al., 2014; Sousa et al., 2016). Therefore, potential strains are commonly excluded by this screening. Another selection methodology is screening in a liquid culture medium. This methodology is currently accepted as the most reliable method, but it also presents inconsistencies as a colorimetric method. The most widespread protocol for solubilizing strains in a liquid medium for determining free P is described by Murphy and Riley (1962). This protocol is based on the reaction of an ascorbic acid (vitamin C) stock solution with added molybdate, forming a blue phosphomolybdenum complex proportional to the amount of free P in the sample. Nonetheless, this method is highly susceptible to chemical interference from reducing agents present in the culture medium, such as ascorbate, which affects the formation of the blue complex and leads to under- or over-estimated free P concentrations (Jarvie et al., 2002; Kowalenko and Babuin, 2007; Kowalenko, 2008; Nagul et al., 2015; Anschutz and Deborde, 2016).

In this study, tests were performed using CaHPO_4_ as a phosphate source, and the hydroponic method was compared with classic methodologies in solid and liquid media, for the selection of phosphate-solubilizing strains. The effects of the strains on plant growth promotion were evaluated. The methodology was tested using *Glycine max* plants as a model because their seedlings are easy to obtain and because of the current need for developing biotechnologies for the cultivation of this oilseed. In addition, the growth-promoting potential of the tested strains was compared with that of the commercial product Biomaphos®, which consisted of a mixture of *Bacillus megaterium* and *B. subtilis* bacteria. We hypothesized that the hydroponic system could be more efficient in selecting phosphate-solubilizing strains, which are plant growth promoters, independent of other nutrients. The hydroponic system requires the presence of a root system, which provides data related to the effect of the isolates on growth promotion and photochemical and photosynthetic performance. Furthermore, the effectiveness of this system was proposed based on the greater induction of plant-microorganism interactions, since in the water-plant condition, root exudates are easily adsorbed in the solution (Hoffland et al., 2006) and are freer to stimulate the tested microorganisms. This study proposed a more efficient approach for selecting strains with the potential to satisfy the current demand for bio-inputs applied to P availability in important crops, such as soybeans.

## Materials and Methods

### Microbial Strains

The potential of eight rhizospheric or endophytic microbial strains was evaluated, with six previously isolated from *Hymenaea courbaril* (for further information see Rocha et al., 2020) and two from Arecaceae *Butia purpurascens* (for further information see da Silva et al., 2018) (Table 1). These strains currently belong to the microorganism collection of the Agricultural Microbiology Laboratory of the IFGoiano Rio Verde campus, Brazil. The potential of these strains was compared with that of the commercial product Biomaphos® (BIOMA, Brazil), which consisted of a mixture of BRM034840 and BRM033112-B strains from *B. megaterium* and *B. subtilis*. The bacterial strains were reactivated in nutrient agar medium (3 g of meat extract, 5 g of peptone, 25 g of agar, and H_2_O qs 1 L) for 48 h at 35°C in an incubation chamber, and then reactivated in potato dextrose agar (PDA; infusion of 200 g of potato, 20 g of dextrose, and 15 g of agar) for 7 days at 35°C in an incubator.

**Table 1:** Microbial strains were used to compare experimental methodologies for the evaluation of the solubilization capacity of calcium phosphate (CaHPO_4_).

**Strain**	**Type**	**Strain code**
*Penicillium sheari*	Fungus	HSCR15-F(SC15)
*Epicoccum keratinophilum*	Fungus	HSCR4-F(SC4)
*Hamigera insecticola*	Fungus	BP33EF-F(33EF)
*Codinaeopsis* sp.	Fungus	BP328EF-F(328EF)
*Bacillus cereus*	Bacterium	HSCE5-B(SC5)
*Bacillus thuringiensis*	Bacterium	HSCR10-B(SC10)
*Paenibacillus alvei*	Bacterium	HPAR12-B(PA12)
*Lysinibacillus fusiformis*	Bacterium	HPAR26-B(PA26)
*Bacillus megaterium* and *Bacillus subtilis*	Bacteria	BRM034840 e BRM033112-B (Biomaphos®)

*In the strain codes HSCR and HPAR, isolated from Hymenaea courbaril; BP, isolated from Butia purpurascens; E, endophytic; R, rhizospheric*.

### Qualitative Assessment of the Solubilization Capacity of CaHPO_4_ in Solid Medium

Bacterial and fungal strains were inoculated in Petri dishes containing GELP culture medium (10 g glucose, 5 g peptone, 0.05 g yeast extract, and 15 g agar). We added 25 ml of calcium dichloride (CaCl_2_) (10%) and 12.5 ml of dipotassium hydrogen phosphate (K_2_HPO_4_) (10%) to form an inorganic phosphate precipitate from CaHPO_4_ (10%), as described by Sylvester-Bradley et al. (1982). The ability of the microorganism to solubilize CaHPO_4_ was confirmed by observing a clear halo around the colony of the bacteria or fungus, in contrast to the opaque medium (Souchie et al., 2007). Plates containing GELP culture medium with CaHPO_4_ added without inoculum were used as negative controls for solubilization.

The capacity of the strains to solubilize CaHPO_4_ was compared by measuring the diameters of the solubilization zones (halos) around the colonies after 7 days of incubation. The solubilization index (SI) was calculated using the method proposed by Berraquero et al. (1976):


$$\begin{array}{l} \text{SI}=\frac{\left(\text{total diameter including colony and halo}\right)}{\left(\text{colony diameter}\right)} \end{array}$$

The SI of the strains was classified according to the methods of Silva Filho and Vidor (2000) as low (SI < 2), medium (2 < SI < 3), or high (SI > 3). The test was conducted in triplicate for each strain tested.

### Quantitative Assessment of CaHPO_4_ Solubilization in Liquid Medium

For this test, bacterial samples were grown under constant agitation using an orbital shaker (NT 712, NOVA TÉCNICA, Brazil) rotating at 90 rpm for 24 h at 30°C in 7 ml of liquid GL culture medium (10 g glucose, 2 g yeast extract). Then, 3 ml of the samples were aseptically removed from each culture to determine the optical density (OD) at 600 nm. All bacterial samples had their OD adjusted to 0.1 by dilution with saline solution (0.85%). Fungal samples were grown in a PDA medium for 4 days at 30°C. CaHPO_4_ solubilization in liquid medium was quantified by inoculating 1 ml of the previously standardized bacterial culture in 10 ml of liquid GL medium, with 1.26 g L^−1^ of the CaHPO_4_ phosphate source. For the evaluation of fungi, 5 mm diameter disks with mycelial growth were removed, which were inoculated in 10 ml of medium (one disk per glass). The cultures were agitated at 90 rpm and 30°C for 72 h. The test was performed in triplicate for each strain tested, using GL medium without inoculum as a negative control. After growth, the pH was measured, and the amount of inorganic P was determined using the vitamin C colorimetric method at 725 nm, according to the methods of Gadagi and Sa (2002).

The *in vitro* solubilization experiments were conducted in a completely randomized design, considering nine treatments with microorganisms (eight strains + Biomaphos®) and a control treatment (without inoculation). CaHPO_4_ solubilization means and pH were subjected to a one-way ANOVA to evaluate only the treatment effect. When significant, the effects were evaluated using the Scott-Knott test at 5% significance probability.

### *In vivo* Assessment of the Solubilizing Potential of CaHPO_4_ Using a Hydroponic System

#### Inoculum Preparation

Bacterial inoculates were prepared in nutrient broth for 24 h at 30°C under constant agitation at 90 rpm. The cell concentration in each culture was estimated by CFU counting in nutrient agar and was standardized to 10^4^ ml^−1^ using 0.85% saline solution. The fungal mycelia were cultivated in a PDA medium for 14 days at 30°C. Subsequently, the plates were superficially washed using 10 ml of 0.85% saline solution per plate, and the spore concentration was determined by counting them in a Neubauer chamber (hemocytometer) using an optical microscope (BA210, MOTIC, Canada) (40–100 × magnification). The spore concentration of the cultures was adjusted to 10^5^ ml ^−1^.

#### Root Colonization and Microscopic Examination

This test was conducted to verify the colonizing potential of the strains to be tested in a hydroponic system. The test was conducted using *G. max* seeds of the Bonus 8579 RSF IPRO cultivar (BRASMAX, Brazil). The seeds were disinfected to remove epiphytes, successively washed in running water, and agitated in water and neutral detergent (Tween) for 5 min. Then, the seeds were immersed in 70% ethanol for 1 min, followed by immersion in sodium hypochlorite (2.5% active chlorine) for 1 min and 30 s, and then again in 70% ethanol for 30 s. Finally, the seeds were washed three times in autoclaved distilled water and planted in plastic trays containing autoclaved sand as substrate, where the seedlings remained for 7 days in a BOD Camera (SSBODU320, PROLAB, Brazil) at 25°C (77F) and 12/12 h photoperiod. During this period, the seedlings were aseptically watered once a day with sterilized water. The plants were then carefully removed from the sand, and the roots were detached and washed four times with sterilized distilled water under vigorous agitation. The roots were then submerged in the previously prepared inoculum solutions and deposited in Petri dishes containing GELP medium. Plates containing roots immersed in autoclaved distilled water were used as a negative control and incubated at 28°C for 48 h. Subsequently, the roots were prefixed with glutaraldehyde, post-fixed with osmium tetraoxide, dehydrated in an ethanol series, transferred to amyl acetate, and critically dried in a dryer with carbon dioxide (CO_2_), following the methodology described by Ghosh et al. (2016). Then, they were coated with gold using an ion jet to evaluate microbial colonization under a scanning electron microscope (Jeol JSM-IT300LV, JEOL USA, Inc., Peabody, MA, US).

#### Experiment in the Hydroponic System

The experiment was conducted in a greenhouse at the Plant Tissue Culture Laboratory of the IFGoiano Rio Verde campus (17° 48' 15.9' ‘S – 50° 54' 19,5” W), from July to August 2020, under an average temperature of 26.85°C and relative humidity of 23.7%. Soybean seeds of the same cultivar were subjected to the same disinfestation and germination treatment in autoclaved sand, where the seedlings remained until they reached a mean size of 15 cm, and then transferred to 4 L pots containing the hydroponic nutrient solution proposed by Hoagland and Arnon (1950) with half ionic strength for 15 days of adaptation. After this period, the plants were subjected to a nutrient solution with full ionic strength.

The inoculation of microorganisms occurred during phase R5 of soybean development. For this, 10 ml of the previously prepared inoculate solutions were added to the nutrient solution along with 10 g L^−1^ of aseptic glucose to stimulate microbial growth in the nutrient solution. Biometric, physiological, and anatomical evaluations were conducted after 10 days of exposure to the tested strains. Plants grown in a nutrient solution without adding microorganisms and grown in a nutrient P-free solution (without the addition of CaHPO_4_) were used as control treatments.

The *in vivo* experiment was conducted in randomized blocks, with nine treatments with microorganisms (eight strains + Biomaphos®) and two control treatments (plants grown in a nutrient solution without microorganisms and plants grown in P-free nutrient solution). All treatments were evaluated in five repetitions, with each repetition consisting of two plants per pot. The results obtained for biometric analyses, tissue P content, and physiological and anatomical analyses were subjected to ANOVA to evaluate the treatment effect. When significant, the effects were evaluated using the Scott-Knott test at 5% probability.

Subsequently, all variables were evaluated using a correlation matrix and combined in a principal component analysis (PCA). Since these variables had different measurement units, a correlation PCA was performed which was constructed using standardized data with a mean of 0 and a standard deviation of 1. The number of components was chosen as a function of the eigenvalues (>1) and explained variance (above 80%). The variables were also evaluated using Pearson's correlation coefficient, and the strength of the relationship was analyzed through R-values and the significance of the interaction at 5% probability. All statistical tests were performed in the R software version 4.0.4 (R Core Team, 2021), using the “ExpDes.pt” (Ferreira et al., 2014) and “FactoMineR” (Husson et al., 2010) packages.

#### Biometric and Tissue P Content Evaluations

The biometric variables plant height, stem diameter (SD), root length (RL), number of leaves (LN), and biomass were evaluated. For biomass evaluation, the plants were segmented into leaves, stems, and roots, and the material was dried in a forced-air oven at 65°C to a constant mass. Subsequently, the dry mass (DM) of each plant part was determined. The sum of the values corresponding to the biomass of each structural component of the plants (LDM + SDM + RDM) enabled the determination of the total DM (TDM).

Leaves, stems, and roots were oven-dried at 65°C until they reached a constant weight (Oven; SL-102/1152, SOLAB, Brazil) and crushed in a Willey mill with a 20 mm-mesh sieve (R-TE-680, TECNAL, Brazil). Finally, the P content was estimated according to the procedure described by Malavolta et al. (1997).

#### Anatomical Evaluation of the Root

The diameter of the root pot elements was evaluated as an indication of root development because it is commonly associated with P availability to plants (Rosolem and Marcello, 1998). Thus, root samples were fixed in glutaraldehyde solution (2.5%) and paraformaldehyde (4%) in sodium cacodylate buffer (pH 7.2) and added to 5 mM calcium chloride (Karnovsky, 1965). The roots were cross-sectioned into 5-μm-thick slices and stained with toluidine blue at pH 6.8 (O'Brien et al., 1964). In these sections, the diameter of the vessel elements was measured using the Anati Quanti 2 software (Aguiar et al., 2007). Each repetition was measured 15 times, and the treatments were evaluated in quintuplicate.

#### Gas Exchange

Gas exchange was evaluated from 7:00 AM to 10:00 AM on the third leaf counted from the apex of the plant using an infrared gas analyzer with a fluorometer (LI-6800xt, LI-COR Inc., Lincoln, USA) and photosynthetically active radiation (1,000 μmol photons m^−2^ s^−1^) at a block temperature of 27°C and relative humidity of ~70%. The net photosynthesis rate (*A*) (μmol CO_2_ m^−2^ s^−1^), stomatal conductance (g_sw_) (mol of H_2_O m^−2^ s^−1^), transpiration (*E*) (H_2_O m^−2^ s^−1^), and internal carbon concentration (*Ci*) (mmol m^−2^ s^−1^) were measured (see Hunt, 2003).

#### Photosynthetic Pigments

The concentration of photosynthetic pigments was evaluated using leaf disks (three fresh matter mass disks of 5 mm diameter each). These disks were covered with a DMSO solution and saturated with calcium carbonate (CaCO_3_) (Santos et al., 2007). Subsequently, they were stored in tubes wrapped in Al foil for 24 h at 65°C. The absorbance of the obtained extract was determined by spectrophotometry at 664, 649, and 480 nm. Chlorophyll *a, b*, and total and carotenoid concentrations were determined according to the methods described by Wellburn (1994).

#### Chlorophyll *a* Fluorescence

Chlorophyll *a* fluorescence OJIP transient was determined using a portable FluorPen FP 100 fluorometer (Photon Systems Instruments, Drasov, Czech Republic). The third leaf of all sample units was previously dark-adapted for 30 min for complete oxidation of the photosynthetic electron transport system. They were later subjected to a 3,000 μmol m^−2^ s^−1^ blue light pulse, with minimum fluorescence (F_0_) and maximum fluorescence (F_M_). These values were used to estimate several PSII bioenergetic indices, according to the methods described by Strasser et al. (2000). The values of the specific light absorption flow per reaction center (ABS/RC), energy flow captured per reaction center at *t* = 0 (TRo/RC), electron transport flow per reaction center (ETo/RC), specific energy dissipation flow at the level of the antenna complex chlorophylls (DIo/RC), photosynthetic performance index (Pi_Abs) that incorporates energy cascade processes from the first absorption events to PQ reduction, the maximum quantum yield of primary photochemistry (PHI_Po), the probability of an exciton moving an electron through the electron transport chain after quinone (PSI_O), and the quantum yield of electron transport (PHI_Eo) after dark adaptation of the leaves (30 min) were determined in this study.

Chlorophyll *a* fluorescence in *G. max* leaves was also evaluated using the modulated Imaging-PAM fluorometer to obtain fluorescence images. Initially, the initial fluorescence (F0) and maximum fluorescence (Fm) were determined in leaves pre-adapted to the dark for 30 min. Then, it was possible to calculate the potential quantum yield of photosystem II (PSII) (Genty et al., 1989). Sequentially, the fluorescence in the light-adapted sample before the saturation pulse (F) and Fm in a light-adapted sample (Fm') were used to obtain the effective quantum yield of photochemical energy conversion in PSII, ΦII = (Fm′-F)/Fm′.

## Results

### *In vitro* Experiment: Solid Culture Medium

In a solid medium, only the *B. thuringiensis* (SC10), *B. megaterium, B. subtilis* (Biomaphos®), and *B. cereus* (SC5) strains demonstrated potential for CaHPO_4_ solubilization through the production of solubilization halos. The solubilizing capacity of these strains was classified as low (SI < 2) (Table 2).

**Table 2:** Evaluation of calcium phosphate (CaHPO_4_) solubilization in solid medium (GELP) by rhizospheric or endophytic bacterial and fungal strains from *Hymenaea courbaril* or endophytic bacterial and fungal strains from *Butia purpurascens*.

**Microorganisms**	**Solubility index**	**Solubilization capacity**
SC10	1.25 a	Low
PA12	NS	NS
Biomaphos®	1.27 a	Low
SC5	1.14 a	Low
SC15	NS	NS
PA26	NS	NS
SC4	NS	NS
33EF	NS	NS
328EF	NS	NS
Control	NS	NS

*Means followed by the same lowercase letter in the column do not significantly differ according to the Scott-Knott test at 5% probability. NS, did not form a solubilization halo*.

### *In vitro* Experiment: Liquid Culture Medium

*B. thuringiensis* (SC10), *Paenibacillus alvei* (PA12), and *B. cereus* (SC5) strains showed the highest solubilization efficiency in a liquid medium containing CaHPO_4_ as a phosphate source. All microbial strains reduced the pH of the medium compared with the control without inoculation, except for fungal *Hamigera insecticola* (33EF) and *Codinaeopsis* sp. (328EF) strains, which presented the lowest solubilization means (Table 3).

**Table 3:** Evaluation of calcium phosphate (CaHPO_4_) solubilization in liquid medium (GL) by rhizospheric or endophytic bacterial and fungal strains from *Hymenaea courbaril* or endophytic bacterial and fungal strains from *Butia purpurascens*.

**Treatments**	**pH**	**Soluble P (mg L^**−1**^)**
SC10	4.79 c	5.53 a
PA12	4.80 c	5.62 a
Biomaphos®	4.89 c	4.61 b
SC5	4.96 b	5.13 a
SC15	5.28 b	3.15 c
PA26	5.48 b	3.69 b
SC4	5.53 b	3.18 c
33EF	6.37 a	1.82 d
328EF	6.49 a	1.48 d
Control	6.73 a	0.33 e

*Means followed by the same lowercase letter in the column did not differ significantly according to the Scott-Knott test at 5% probability*.

### Root Colonization

**Figure 1:**
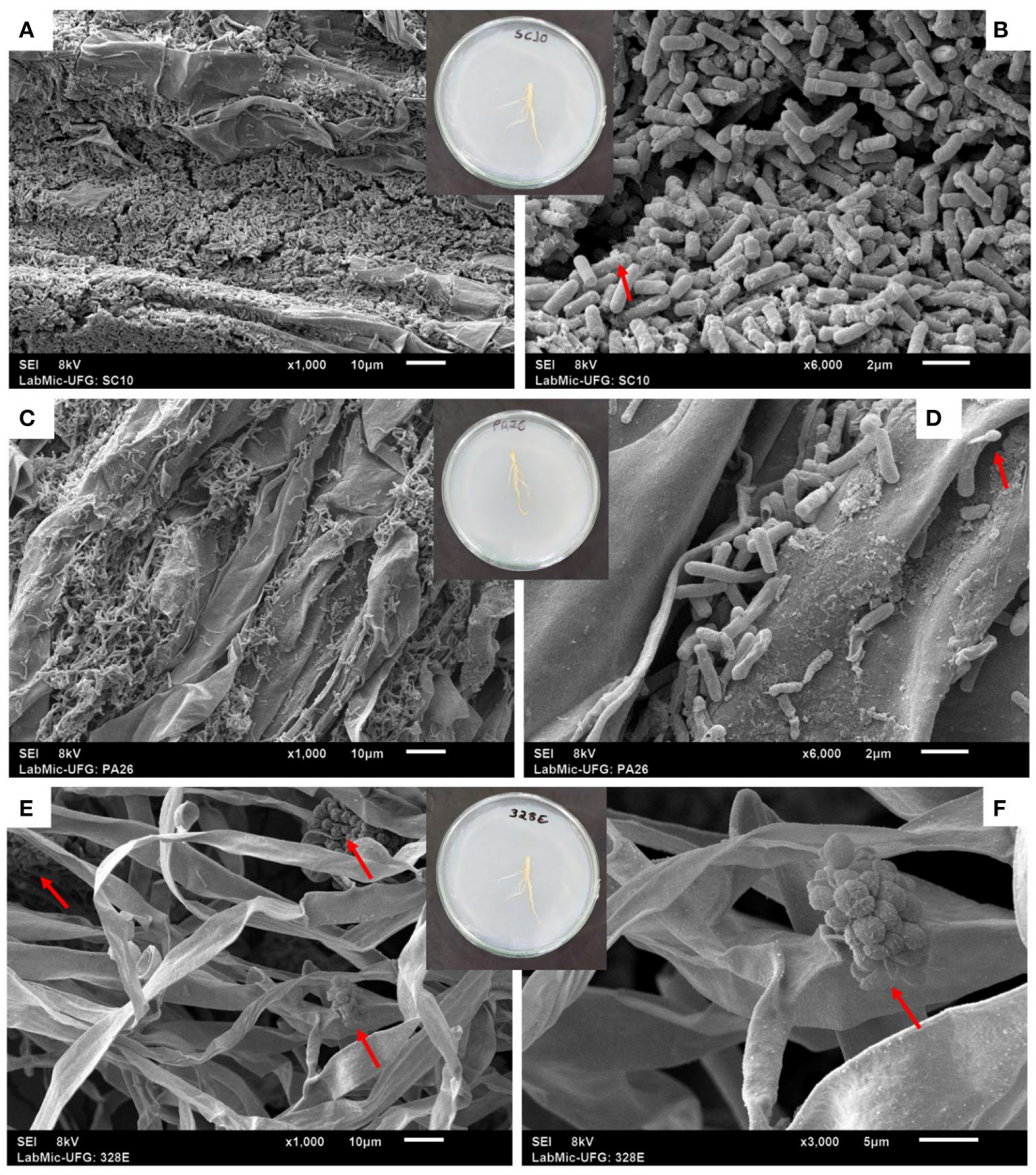
Microscopic aspects of root colonization in soybean plants (*Glycine max)* treated with the bacterial strains SC10 = *Bacillus thuringiensis* **(A,B)** and PA26 = *Lysinibacillus fusiformis* **(C,D)**, and the fungal strain 328EF = *Codinaeopsis* sp. **(E,F)**. Red arrows indicate the presence of protein crystals **(B)** and spores **(D–F)**.

All bacteria and fungi evaluated demonstrated the ability to effectively colonize the root system, forming large superficial aggregates (Supplementary Figures 1, 2). Electron microscopy analyses showed the formation of protein crystals in *B. thuringiensis* (SC10) colonies and bacterial spores in *Lysinibacillus fusiformis* (PA26) root colonies (Figures 1A–D). They also found a large concentration of fungal spores in *Codinaeopsis* sp. (328E) colonies (Figures 1E,F).

### *In vivo* Experiment: Hydroponic Cultivation

#### Evaluation of Growth Promotion

The exposition of *G. max* seedlings to the microorganisms tested did not affect the height of the plants; the means ranged between a minimum of 84.8 cm in plants inoculated with *Epicoccum keratinophilum* (SC4) and a maximum of 93.8 cm in plants treated with *P. alvei* (PA12). However, the development of plants in a control solution without P was significantly affected by the unavailability of this element, with a mean height for these plants of only 64.25 cm (Supplementary Figure 3A). Similar results were obtained for RL, which remained statistically equal in plants inoculated with different microorganisms and in a solution without microorganisms, with the means ranging between a minimum of 46.5 cm in plants inoculated with *E. keratinophilum* (SC4) and without microorganisms and a maximum of 93.8 cm in plants treated with *P. alvei* (PA12). Plants kept in a nutrient solution without P also had their RL affected, with a mean of 34 cm (Supplementary Figure 3B).

**Figure 2:**
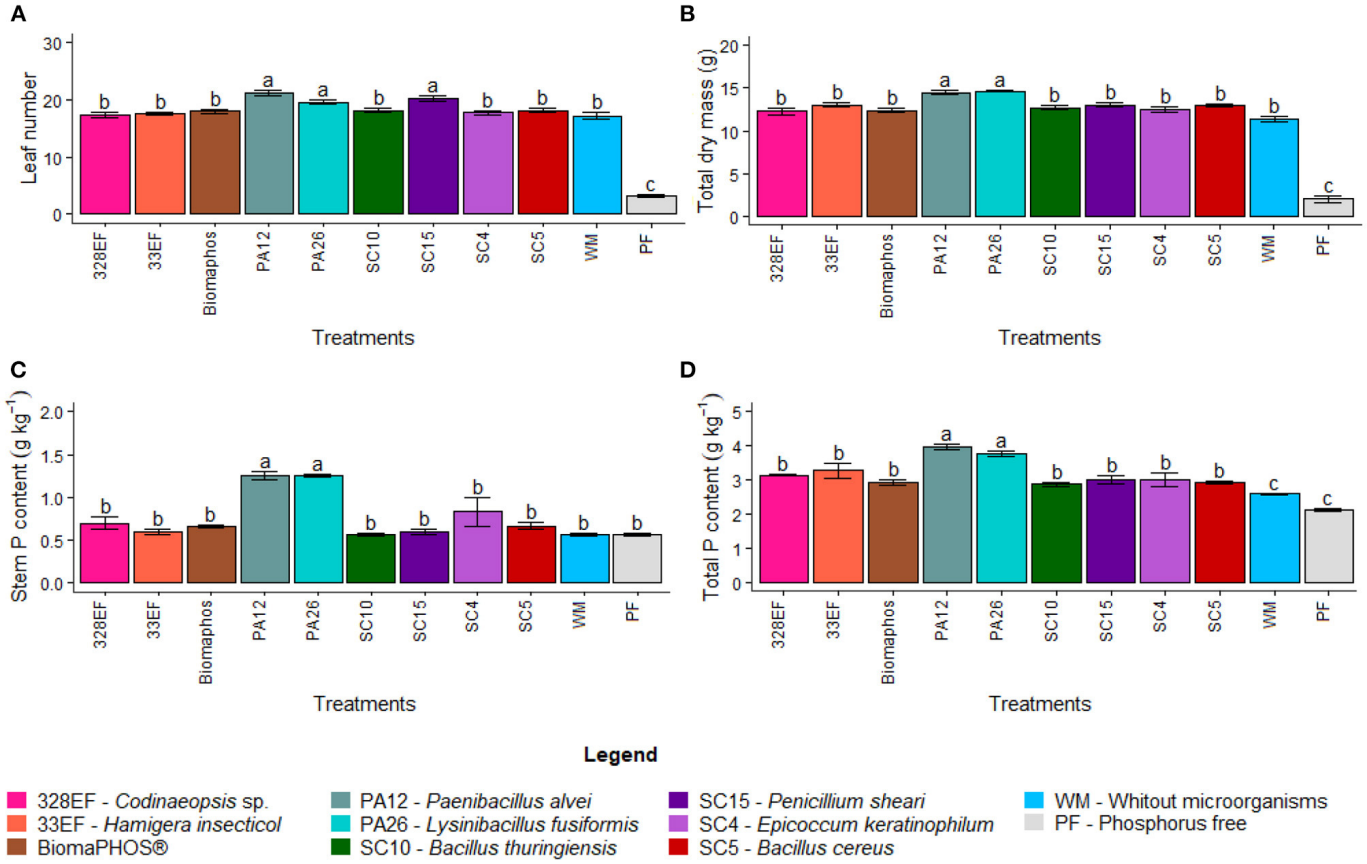
Number of leaves **(A)**, total dry mass **(B)**, P tissue content in the stem **(C)**, and total P tissue content **(D)** in soybean plants (*Glycine max)* grown in nutrient solution providing calcium phosphate (CaHPO_4_) as the phosphate source and inoculated with four bacterial strains and four fungal strains. Means followed by the same lowercase letter do not differ from one another according to the Scott-Knott test at 5% probability.

Standard deviation means also followed the same pattern: not differing between plants grown in a solution without microorganisms and inoculated plants, ranging from a minimum of 0.38 mm in non-inoculated plants to a maximum of 0.46 mm in plants treated with *P. alvei* (PA12). As described for the previous variables, plants subjected to a nutrient solution without P also presented low SD development (0.16 mm) (Supplementary Figure 3C). However, the LN was significantly affected by the inoculation treatments, with the highest values for plants inoculated with *P. alvei* (PA12), *L. fusiformis* (PA26), and *Penicillium sheari* (SC15) (21.2, 20.2, and 19.6 leaves, respectively). The absence of P in the solution also affected the LN in the plants, with a mean of only 3.25 leaves in plants developed with this treatment (Figure 2A).

The dry matter mass of leaves, stems, and roots did not differ between plants inoculated with microorganisms and plants grown in a solution without microorganisms. Leaf dry matter means varied between a minimum of 4.39 g in non-inoculated plants and a maximum of 5.2 g in plants treated with *L. fusiformis* (PA26) (Supplementary Figure 4A). Shoot dry matter mass followed a similar trend, ranging from a minimum of 4.05 g in non-inoculated plants and a maximum of 5.31 g in plants treated with *L. fusiformis* (PA26) (Supplementary Figure 4B). Root dry matter also followed the pattern described above, ranging from a minimum of 2.91 g in non-inoculated plants to a maximum of 4.12 g in plants treated with *L. fusiformis* (PA26) (Supplementary Figure 4C). However, plants grown in a solution without P presented the lowest values for LDM, SDM, and RDM (0.196, 1.036, and 0.847 g, respectively).

For TDM, the highest values were observed for plants inoculated with *P. alvei* (PA12) and *L. fusiformis* (PA26) (14.63 and 14.43 g, respectively). Plants grown in solution without microorganisms had a mean of 11.35 g TDM, but plants kept in solution without P had the lowest values (2.08 g) (Figure 2B).

#### P Absorption Performance Evaluation in P Tissue Content

Phosphorus content did not differ in leaves of plants subjected to the different treatments evaluated, and the means ranged between a minimum of 0.67 g in plants grown without P and a maximum of 1.30 g kg^−1^ in plants treated with *P. alvei* (PA12) (Supplementary Figure 5A). Stem P content was affected by the inoculation treatments, being higher in plants inoculated with *P. alvei* (PA12) and *L. fusiformis* (PA26) (1.25 and 1.25 g kg^−1^, respectively) (Figure 2C). The P content in roots was higher for plants inoculated with *Codinaeopsis* sp. (328EF), *H. insecticola* (33EF), *P. alvei* (PA12), *L. fusiformis* (PA26), *B. thuringiensis* (SC10), and *P. sheari* (SC15) (1.50, 1.57, 1.40, 1.45, 1.35, and 1.35 g kg^−1^, respectively) (Supplementary Figure 5B). The evaluation of total P content showed the highest means in plants inoculated with the bacteria *P. alvei* (PA12) and *L. fusiformis* (PA26) (3.95 and 3.77 g kg^−1^), while the lowest means were verified in plants growing in a solution without microorganisms (2.58 g kg^−1^) and P-free (2.13 g kg^−1^) (Figure 2D).

#### Photosynthetic Performance Assessment: Gas Exchange

**Figure 3:**
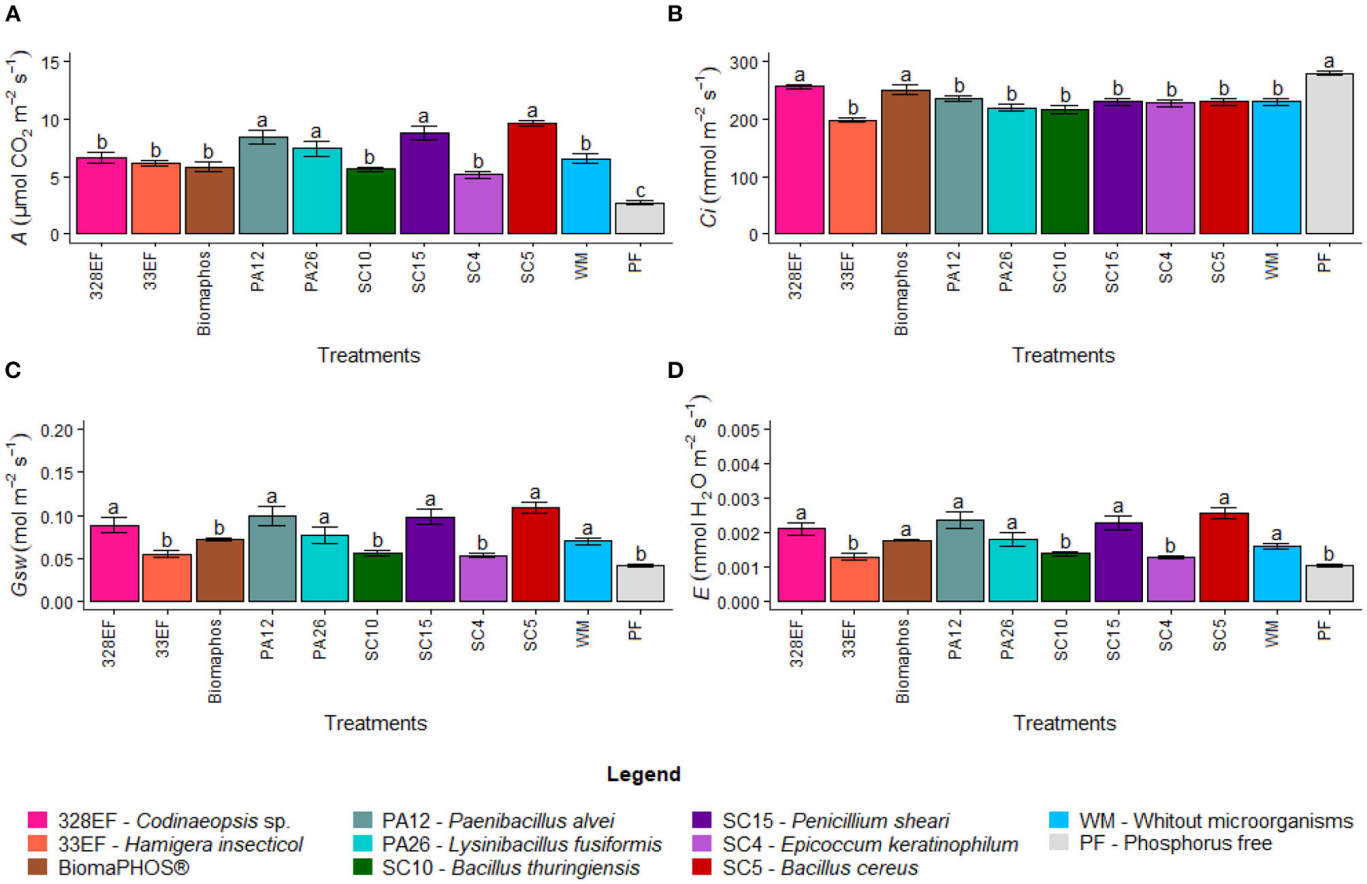
Photosynthetic rate (*A*) **(A)**, internal CO_2_ concentration (*Ci*) **(B)**, stomatal conductance (*Gsw*) **(C)**, and transpiration rate (*E*) **(D)** in soybean plants (*G. max)* grown in nutrient solution providing calcium phosphate (CaHPO_4_) as a phosphate source and inoculated with four bacterial strains and four fungal strains. Means followed by the same letter do not differ from one another according to the Scott-Knott test at 5% probability.

Microbial inoculation significantly affected gas exchange rates in *G. max*. The net CO_2_ assimilation rate (*A*) was higher in plants inoculated with the strains *P. alvei* (PA12), *L. fusiformis* (PA26), *B. cereus* (SC5), and *P. sheari* (SC15) at 8.41, 7.43, 9.65, and 8.78 μmol CO_2_ m^−2^ s^−1^, respectively (Figure 3A). However, these plants presented low rates of internal CO_2_ (*Ci*) concentration, respectively 235.56, 219.9, 230.85, and 230.24 mmol m^−2^ s^−1^, while the highest means were verified in plants grown in a solution without P (280.32 mmol m^−2^ s^−1^) and inoculated with *Necropsied* sp. (328EF) and Biomaphos® (256.96 and 251.16 mmol m^−2^ s^−1^) (Figure 3B). A behavior similar to that observed for *A* was observed for stomatal conductance (*Gsw*), with the highest means obtained for inoculation treatments with *Necropsied* sp. (328EF), *P. alvei* (PA12), *L. fusiformis* (PA26), *P. sheari* (SC15), and *B. cereus* (SC5) strains (0.072, 0.099, 0.077, 0.098, and 0.109 mol m^−2^ s^−1^, respectively), and for plants grown in solution without microorganisms (0.07 mol m^−2^ s^−1^) (Figure 3C). The transpiration rate (*E*) was higher in plants grown in a solution without microorganisms (0.0016) and inoculated with *Necropsied* sp. (328EF), Biomaphos®, *P. alvei* (PA12), *L. fusiformis* (PA26), *P. sheari* (SC15), and *B. cereus* (SC5) at 0.0021, 0.0018, 0.0024, 0.0018, 0.0023, and 0.0026 *E*, respectively (Figure 3D).

#### Photosynthetic Performance Assessment: Photosynthetic Pigments

**Figure 4:**
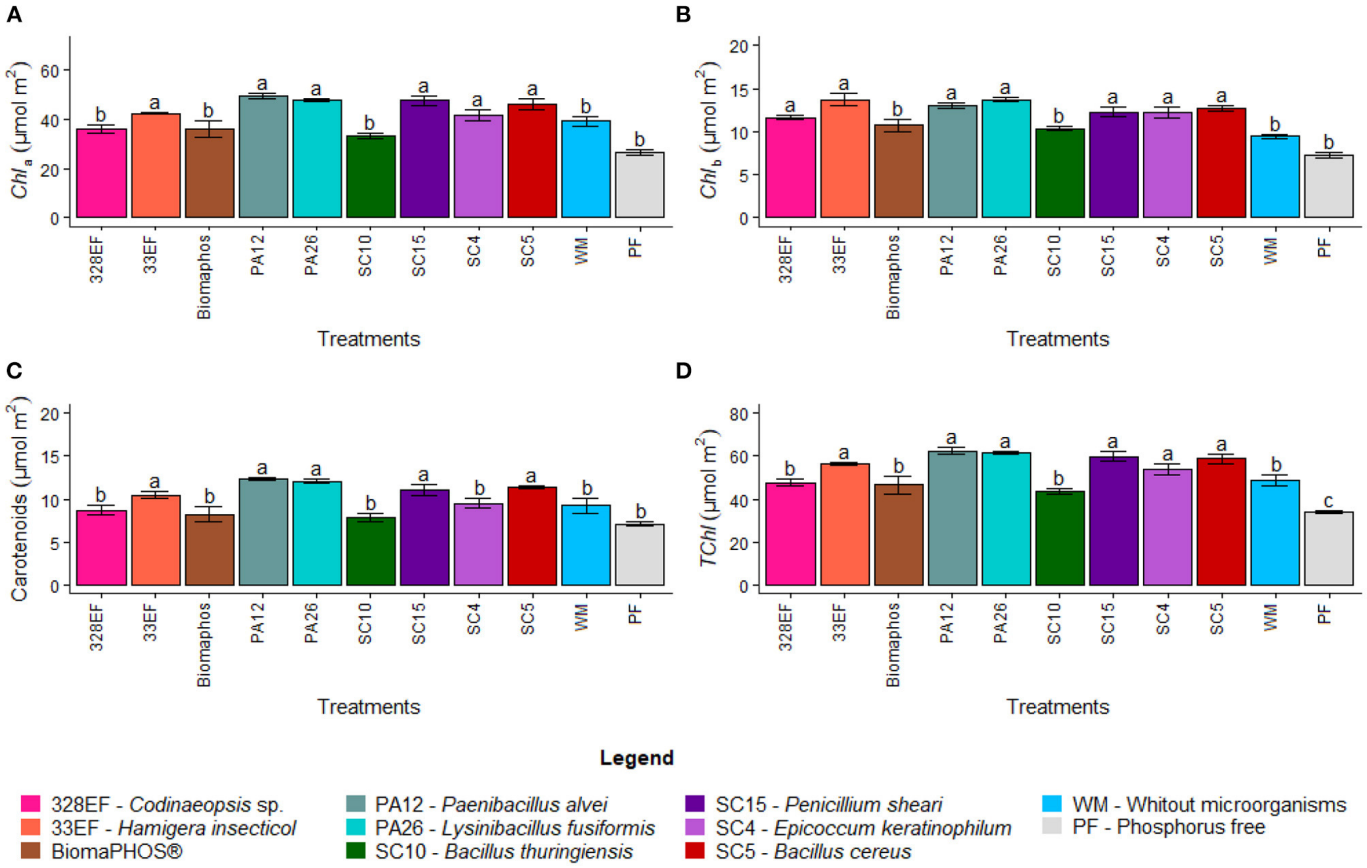
Chlorophyll *a* content **(A)**, chlorophyll *b* content **(B)**, carotenoid content **(C)**, and total chlorophyll **(D)** in soybean plants (*G. max*) grown in nutrient solution providing calcium phosphate (CaHPO_4_) as a phosphate source and inoculated with four bacterial strains and four fungal strains. Means followed by the same letter do not differ from one another according to the Scott-Knott test at 5% probability.

Chlorophyll *a* content increased in plants inoculated with *H. insecticola* (33EF), *P. alvei* (PA12), *L. fusiformis* (PA26), *P. sheari* (SC15), *E. keratinophilum* (SC4), and *B. cereus* (SC5) at 42.437, 49.240, 47.717, 47.571, 41.469, and 46.052 μmol m^−2^, respectively (Figure 4A). Chlorophyll *b* content also increased following a pattern similar to that observed for chlorophyll *a*, but it was higher in plants subjected to inoculation with *Necropsied* sp. (328EF), *H. insecticola* (33EF), *P. alvei* (PA12), *L. fusiformis* (PA26), *P. sheari* (SC15), *E. keratinophilum* (SC4), and *B. cereus* (SC5) strains (11.644, 13.723, 13.069, 13.726, 12.231, 12.269, and 12.704 μmol m^−2^) (Figure 4B).

Microbial inoculation also affected the carotenoid content, which was higher in plants inoculated with *H. insecticola* (33EF), *P. alvei* (PA12), *L. fusiformis* (PA26), *P. sheari* (SC15), and *B. cereus* (SC5), with mean values of 10.458, 12.275, 12.084, 11.034, and 11.382 μmol m^−2^ (Figure 4C). Similar to other chlorophylls, the total chlorophyll content varied, with the highest means obtained in plants treated with *H. insecticola* (33EF), *P. alvei* (PA12), *L. fusiformis* (PA26), *P. sheari* (SC15), *E. keratinophilum* (SC4), and *B. cereus* (SC5) (56.161, 62.309, 61.444, 59.802, 53.738, and 58.756 μmol m^−2^). However, much lower mean values were observed in plants grown in a solution without P (33.832 μmol m^−2^) (Figure 4D).

#### Photosynthetic Performance Assessment: Chlorophyll a Fluorescence

**Figure 5:**
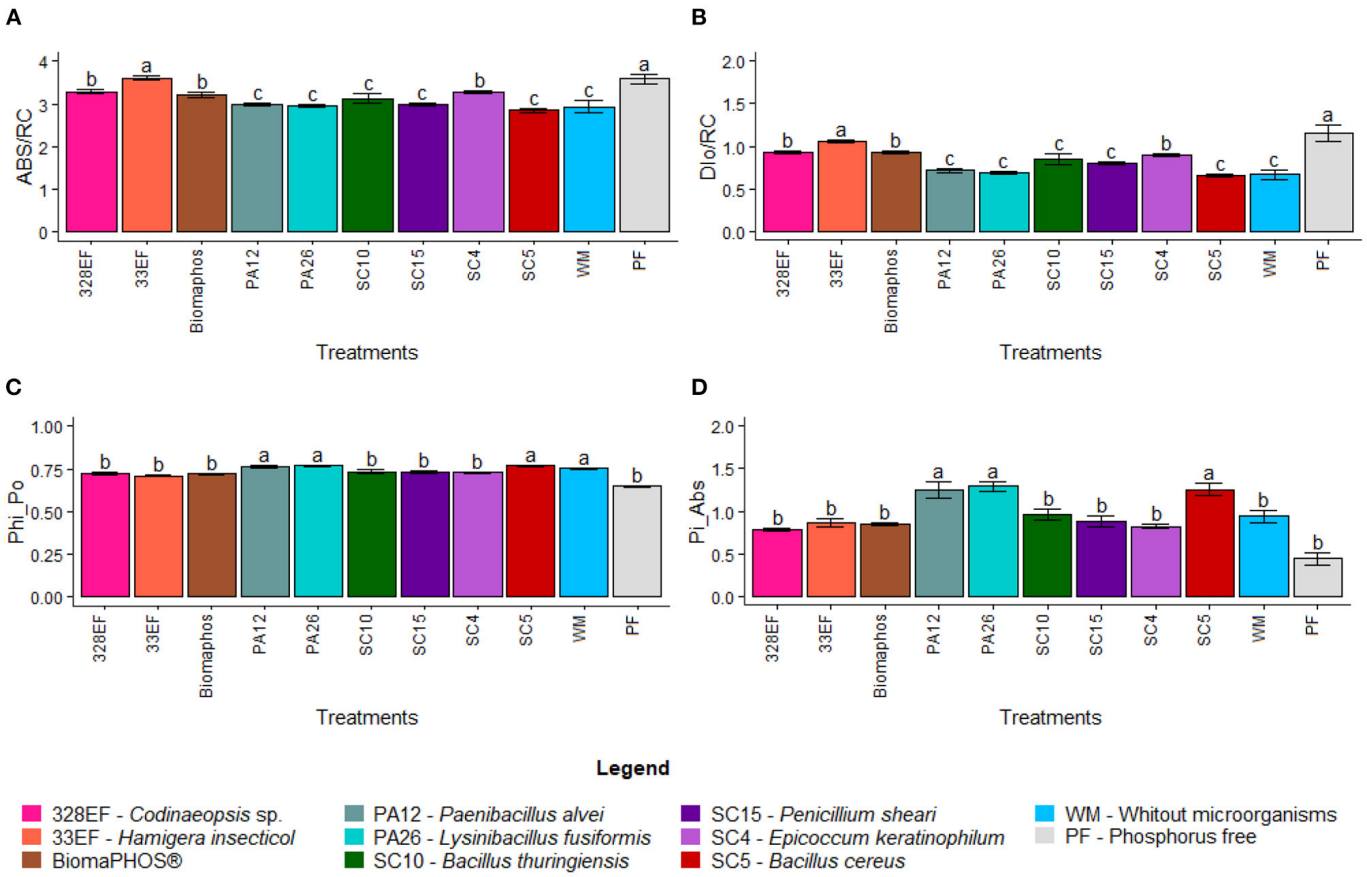
Absorption flow by RC (ABS/RC) **(A)**, specific energy dissipation flow (DIo/RC) **(B)**, the maximum quantum yield of primary photochemistry (PHI_Po) **(C)**, and photosynthetic performance index (Pi_Abs) **(D)** in soybean plants (*G. max)* grown in nutrient solution providing calcium phosphate (CaHPO_4_) as a phosphate source and inoculated with four bacterial strains and four fungal strains. Means followed by the same letter do not differ from one another according to the Scott-Knott test at 5% probability.

As expected, the specific energy flows of the active reaction centers ABS/RC and DIo/RC were similarly affected by microbial inoculation; therefore, the lowest mean ABS/RC values were observed in plants grown with the microorganisms *P. alvei* (PA12), *L. fusiformis* (PA26), *B. thuringiensis* (SC10), *P. sheari* (SC15), and *B. cereus* (SC5) (2.986, 2.962, 3.127, 2.989, and 2.859, respectively), as well as in plants grown in a solution without microorganisms (2.935) (Figure 5A). The lowest mean DIo/RC values were obtained in plants subjected to the same treatment sequence as above (0.714, 0.686, 0.842, 0.796, 0.660, and 0.667, respectively) (Figure 5B). The means observed for TRo/RC and ETo/RC were not affected by any of the evaluated treatments. TRo/RC values ranged from a minimum of 2.193 in plants inoculated with *P. sheari* (SC15) and a maximum of 2.546 in plants treated with *H. insecticola* (33EF), whereas ETo/RC means varied between a minimum of 0.985 in plants grown in nutrient P-free solution and a maximum of 1.384 in plants treated with the fungus *H. insecticola* (33EF) (Supplementary Figures 6A,B).

The kinetic parameters of Chl *a* fluorescence were also significantly affected by inoculation with microorganisms. The maximum quantum yield of primary photochemistry (Phi_Po) was higher for plants inoculated with *P. alvei* (PA12), *L. fusiformis* (PA26), and *B. cereus* (SC5) (0.763, 0.769, and 0.769, respectively), as well as in plants grown in a solution without microorganisms (0.752) (Figure 5C). The probability of an exciton moving an electron through the electron transport chain after quinone (Qa) (Psi_o) was reduced in plants inoculated with *Codinaeopsis* sp. (328EF) (0.443) and under P-free solution (0.406) (Supplementary Figure 6C). The means observed for the quantum yield of electron transport (Phi_Eo) followed the same pattern reported previously for Psi_o, with reduced values under *Codinaeopsis* sp. (328EF) (0.323) and P-free solution (0.277) (Supplementary Figure 6D). The photosynthetic performance index (Pi_ABS) was higher in plants inoculated with *P. alvei* (PA12), *L. fusiformis* (PA26), and *B. cereus* (SC5) (1.244, 1.289, and 1.255), suggesting that these plants have better performance in converting light energy into chemical energy (Figure 5D).

**Figure 6:**
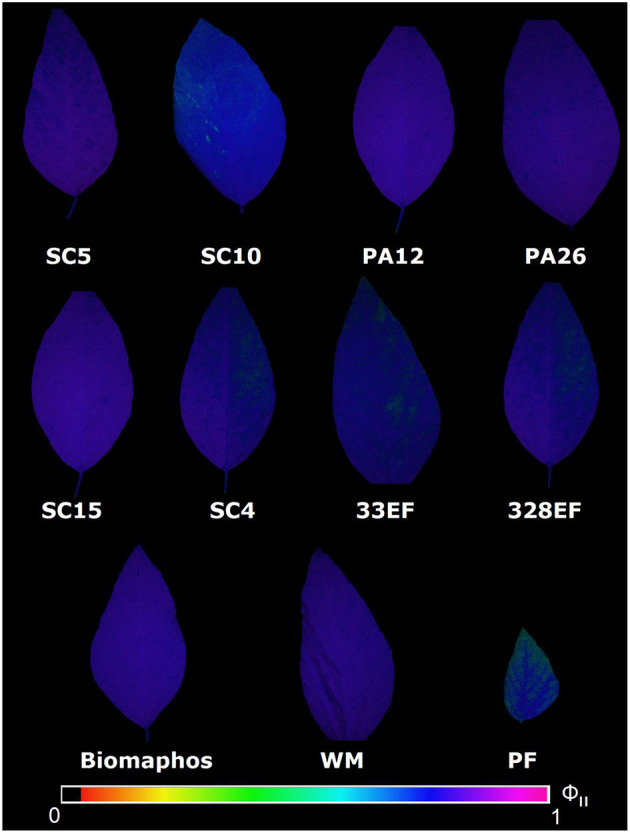
Fluorescence images of chlorophyll-*a* obtained for the effective quantum yield of photochemical energy conversion in PSII (ΦII) in leaves of *Glycine max* grown in nutrient solution providing calcium phosphate (CaHPO_4_) as a phosphate source and inoculated with four bacterial strains and four fungal strains. SC5, *Bacillus cereus*; SC10, *B. thuringiensis;* PA12, *Paenibacillus alvei*; PA26, *Lysinibacillus fusiformis*; SC15, *Penicillium sheari*; SC4, *Epicoccum keratinophilum*; 33EF, *Hamigera insecticola*; 328E, *Codinaeopsis* sp.; Biomaphos®, *B. megaterium* and *B. subtilis*; WM, without microorganisms; PF, phosphorus-free.

Fluorescence imaging analyses confirmed the superiority of the behavioral pattern of chlorophyll *a* fluorescence in plants inoculated with *P. alvei* (PA12), *L. fusiformis* (PA26), and *B. cereus* (SC5) and the inferiority of the pattern observed in plants grown in a P-free solution, as well as in plants inoculated with *E. keratinophilum* (SC4), *Codinaeopsis* sp. (328EF), and *H. insecticola* (33EF) (Figure 6).

#### Assessment of Anatomical Performance: Root Anatomy

**Figure 7:**
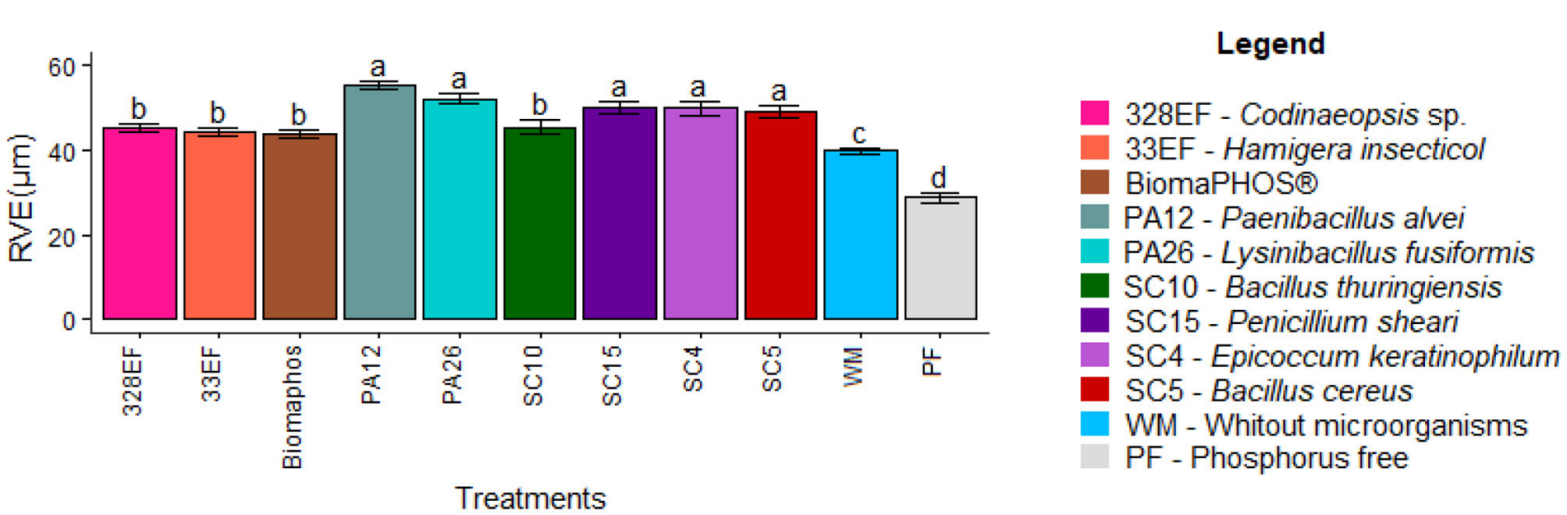
Diameter of root vessel elements (RVE) in soybean plants (*G. max*) cultivated in nutrient solution providing calcium phosphate (CaHPO_4_) as a phosphate source and inoculated with four bacterial strains and four fungal strains. Means followed by the same letter do not differ from one another according to the Scott-Knott test at 5% probability.

The inoculation treatments affected the diameter of the root vessel elements. Therefore, the highest means were observed in plants inoculated with *P. alvei* (PA12), *L. fusiformis* (PA26), *P. sheari* (SC15), *E. keratinophilum* (SC4), and *B. cereus* (SC5) (55.383, 52.223, 49.943, 49.867, and 49.32 μm, respectively). In contrast, the lowest means were observed in plants grown in a P-free solution (28.787 μm), followed by a solution without microorganisms (39.91 μm) (Figure 7).

**Figure 8:**
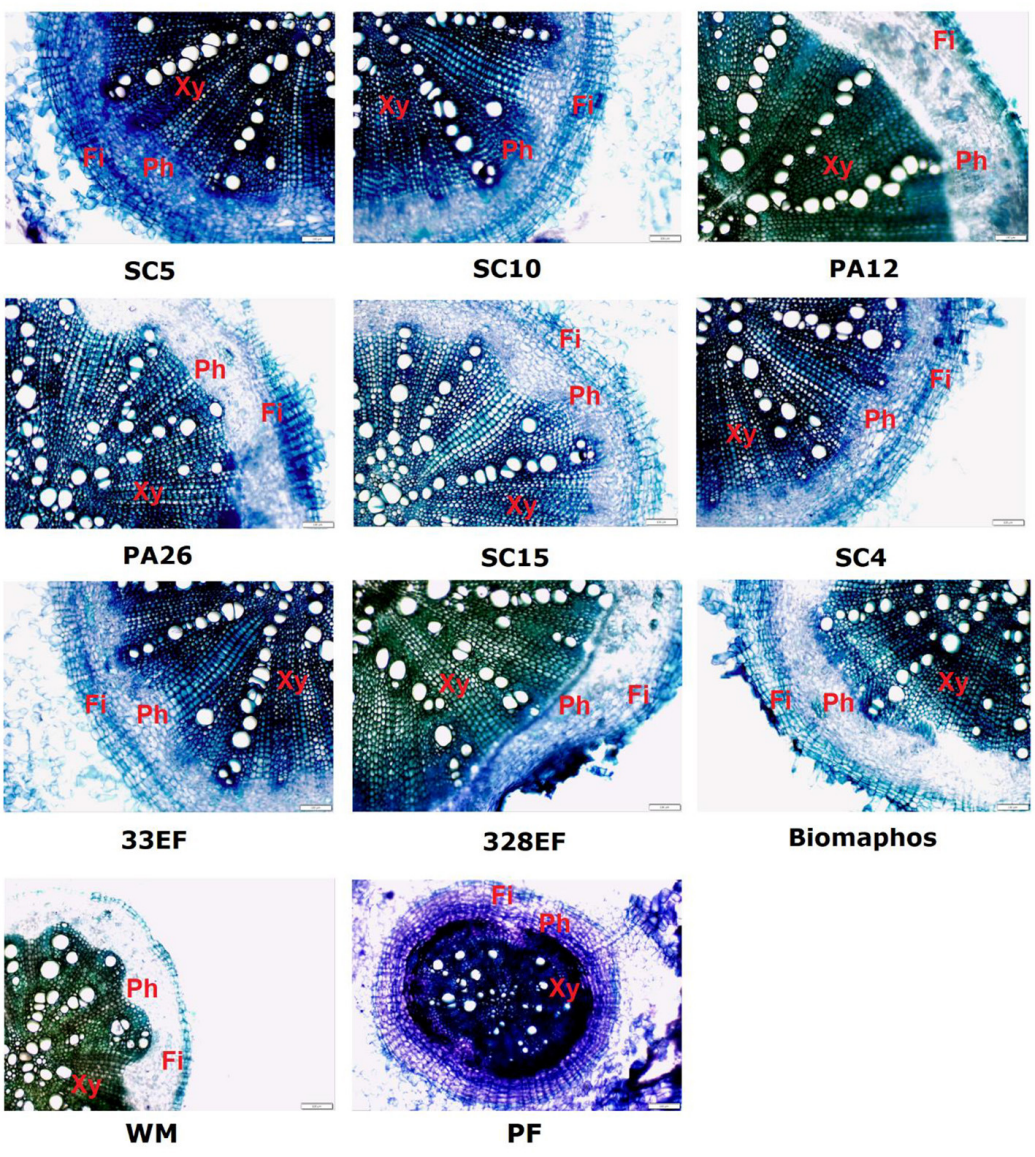
Cross-sections showing different anatomical regions (Xy, Xylem; Ph, Phloem; Fi, Fibers) of soybean roots (*G. max*) grown in nutrient solution providing calcium phosphate (CaHPO_4_) as a phosphate source and inoculated with four bacterial strains and four fungal strains. SC5, *B. cereus*; SC10, *B. thuringiensis*; PA12, *P. alvei*; PA26, *L. fusiformis*; SC15, *P. sheari*; SC4, *E. keratinophilum*; 33EF, *H. insecticola*; 328EF, *Codinaeopsis* sp.; Biomaphos®, *B. megaterium* and *B. subtilis*; WM, without microorganisms; PF, phosphorus-free.

Anatomical analyses showed superior development of vessel elements and root caliber of plants treated with *P. alvei* (PA12) and *L. fusiformis* (PA26) strains. Root development was compromised in plants grown in a P-free solution, as well as in plants not subjected to microbial action (Figure 8).

#### PCA and Correlation Between Variables

**Figure 9:**
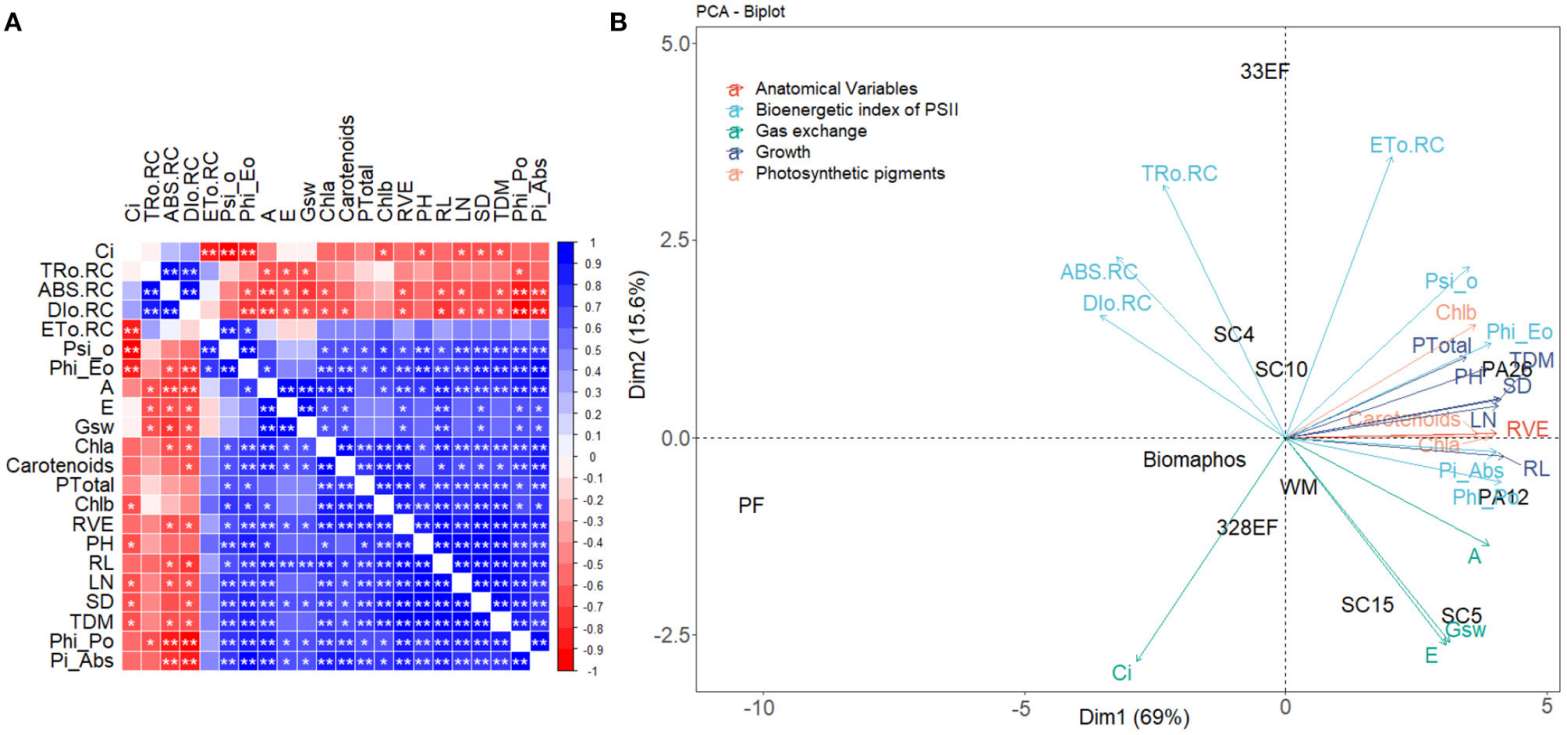
Analysis of the main components of growth variables, photosynthetic pigments, gas exchange, chlorophyll-*a* fluorescence parameters, and anatomical development of root vessel elements **(A)** and correlations between the means observed for these variables **(B)** in soybean plants (*Glycine max*) grown in nutrient solution providing calcium phosphate (CaHPO_4_) as a phosphate source and inoculated with four bacterial strains and four fungal strains. SC5, *B. cereus*; SC10, *B. thuringiensis*; PA1, *P. alvei*; PA26, *L. fusiformis*; SC15, *P. sheari*; SC4, *E. keratinophilum*; 33EF, *H. insecticola*; 328EF, *Codinaeopsis* sp.; Biomaphos®, *B. megaterium* and *B. subtilis*; WM, without microorganisms; PF, phosphorus-free.

The two-dimensional graph of principal components showed a negative correlation between the parameters DIo/RC, ABS/RC, TRo/RC, and *Ci* and the other biometric parameters: fluorescence, photosynthetic pigment content, and gas exchange, signaling the means for plants subjected to *E. keratinophilum* (SC4), *Codinaeopsis* sp. (328EF), Biomaphos®, and *H. insecticola* (33EF); plants grown in P-free solution accounted for most of the variation found in these variables. Thus, these plants were mainly associated with negative photosynthetic performance indices. On the other hand, the means verified in plants inoculated with *P. alvei* (PA12), *L. fusiformis* (PA26), and *B. cereus* (SC5) defined most of the variation in growth parameters, P content, photosynthetic performance, and photosynthetic pigments. These symbiotic microorganisms seem to improve the overall performance of the inoculated *G. max* plants (Figure 9A). The negative correlation between DIo/RC, ABS/RC, TRo/RC, and *Ci* means and the other biometric parameters (fluorescence, photosynthetic pigment content, and gas exchange) was more evident when the graphic pattern of the correlations between all variables was analyzed (Figure 9B).

## Discussion

### Solubilization Results Obtained Using *in vitro* Methodologies Are Not Consistent With the Hydroponic System

Tests conducted in solid medium attested to the solubilizing potential of *B. thuringiensis* (SC10), *B. cereus* (SC5), and Biomaphos®, while in a liquid medium, the capacity was observed for *B. thuringiensis* (SC10), *P. alvei* (PA12), and *B. cereus* (SC5). The superposition of the two strains in these results showed a low coherence between these two methodologies. However, *in vivo* analysis using the hydroponic system showed that *P. alvei* (PA12) and *L. fusiformis* (PA26), strains not shown to solubilize CaHPO_4_ in any of the *in vitro* tests were more efficient at promoting growth and providing free P to the plant, which was demonstrated by the higher total P content in *G. max* tissues. In fact, *in vitro* solubilizing potential assessments were susceptible to the influence of many factors, as they significantly modify the living conditions of microorganisms. The growth temperature, pH, and nutritional composition of the culture medium could affect growth and metabolism (e.g., Gibson and Mitchell, 2004; Walpola et al., 2012; Tan and Ramamurthi, 2014; Yang et al., 2018), including the ability of isolates to produce organic acids or phosphatase enzymes directly associated with the potential of the microorganism to release P from forms where it is immobilized as CaHPO_4_, Ca_3_(PO_4_)_2_, AlPO_4_, and FePO_4_.

In general, solubilization in a liquid culture medium is considered more reliable, as it is a quantitative methodology (Sousa et al., 2016). In this methodology, free P could be quantified, and the access of organic acids and phosphatase enzymes to the phosphate source was more direct. However, the absence of a host and the impossibility of quantifying the actual P absorbed by the plant made this methodology questionable. Therefore, the use of *G. max* as a model for growth, tissue content, and photosynthetic and anatomical performance evaluations characterized *P. alvei* (PA12) and *L. fusiformis* (PA26) strains was more effective in the solubilization of CaHPO_4_ because of its general effects on general growth promotion.

*Paenibacillus* is one of the Firmicutes genera predominantly found in association with plants (Yadav et al., 2017), and the potential of *P. alvei* has already been described in the literature as a disease suppressor and an inducer of crop growth and yield (Schoina et al., 2011; Kumar et al., 2012; Kalaiselvi et al., 2019). *Lysinibacillus* species have also drawn attention as effective bioremediation, biostimulant, and biocontrol agents (Ahsan and Shimizu, 2021). In the studies by Vendan et al. (2010) and He et al. (2014), *L. fusiformis* was described as an effective phosphate solubilizer, although other functional traits have already been associated with this species, such as auxin synthesis and fungal biocontrol (Hanh and Mongkolthanaruk, 2017; Passera et al., 2021). Our work corroborated the direct association between these species and plant growth promotion.

### The Hydroponic System Allows the Association of Phosphate Solubilization With Growth Promotion and Improved Photosynthetic and Anatomical Performance

The inoculation of *P. alvei* (PA12) and *L. fusiformis* (PA26) strains in the nutrient solution resulted in higher P absorption by *G. max* plants, and this better nutritional aspect improved growth, such as increased LN and TDM. Some studies have confirmed that solubilizing strains improve the nutritional status of plants, which results in increased chlorophyll concentrations and improved gas exchange parameters (Singh et al., 2018; Linu et al., 2019; Liu et al., 2020; Borowiak et al., 2021).

Plants inoculated with *P. alvei* (PA12) and *L. fusiformis* (PA26) strains also showed improved photosynthetic rates compared with plants grown in a solution without microorganisms. High *A* values are closely related to high RuBisCO activity (Bowes, 1991). Studies have suggested that an optimal P supply increases RuBisCO concentration and activity in leaves (Warren and Adams, 2002; Lin et al., 2009; e.g., Alori et al., 2017; Wang et al., 2018). Chu et al. (2018) demonstrated that decreased photosynthetic rates in *G. max* could be due to the stress caused by low P availability, as it participates in the structural regulation of the leaf, ATP synthesis, CO_2_ absorption and transport, photosynthetic electron transport, and determination of the levels of enzymes related to the Calvin cycle. Thus, phosphate-solubilizing microorganisms can play an important role in photosynthetic performance by increasing P availability for plants (e.g., Wu et al., 2019; Anbi et al., 2020).

The inoculation of *G. max* with *P. alvei* (PA12) and *L. fusiformis* (PA26) bacteria also resulted in increased concentrations of photosynthetic pigments compared with plants that grew without microbial inoculation or P administration. These solubilizing strains ensured the necessary supply for pigment synthesis by increasing P release and absorption by plants. Some studies have reported that P deficiency decreases chlorophyll synthesis (Choi and Lee, 2012; Viégas et al., 2018), and that root colonization by growth-promoting microorganisms, such as phosphate solubilizers, can increase the synthesis of carotenoids (Vafadar et al., 2014; Chen et al., 2017), and plant resistance to biotic and abiotic stress factors (Vafadar et al., 2014) by protecting the photosynthetic apparatus, reducing photodamage, and photoinhibition effects (Sharma et al., 2015; Uarrota et al., 2018). Furthermore, microorganisms can affect the N_2_ fixation process and the synthesis of growth-promoting substances and other compounds, such as auxins, which also have a positive effect on photosynthetic pigment production (Bashan and De-Bashan, 2010; Ahemad and Kibret, 2014; Santos et al., 2019). Phosphate-solubilizing microorganisms influence some growth-associated compounds, such as cytokinins (Abbamondi et al., 2016; Kudoyarova et al., 2017), which have a positive effect on chlorophyll biosynthesis, delaying senescence, and programmed death processes (Kunikowska et al., 2013; Zwack and Rashotte, 2013; Danilova et al., 2020; Zhang et al., 2021). Jinal et al. (2021) demonstrated that inoculation of maize seeds with *Lysinibacillus* can improve the growth and synthesis of chlorophylls-a and -b. The effects observed on the concentration of photosynthetic pigments in *G. max*, induced by *P. alvei* (PA12) and *L. fusiformis* (PA26) may be associated with the phosphate solubilizer and phytohormone producer potential (Vendan et al., 2010; Hanh and Mongkolthanaruk, 2017) of these species.

In general, plants treated with *P. alvei* (PA12) and *L. fusiformis* (PA26) absorbed more P, which is an adaptive reflection of the increased diameter of RVE. The roots were efficiently colonized by these bacteria. Microbial colonization is commonly related to increased size and density of the primary root and root hairs (Verbon and Liberman, 2016), which are important for increasing P absorption (Gahoonia et al., 2001). However, *in vitro* culture conditions seem to stress *L. fusiformis* (PA26), with evidence of spore formation in the root colonies of this strain. This occurs because when many species of the order Bacillales are subjected to abiotic stress, they tend to sporulate as a resistance mechanism (Paredes-Sabja et al., 2011; Tan and Ramamurthi, 2014). Spore inactivity may explain the inefficiency of this strain in solubilizing CaHPO_4_ in a liquid medium.

### The Hydroponic System Allows Separating Growth-Promoting Action From Biotic Stress Induction Through Photosynthetic Performance

Microbial inoculation affected ABS/RC and DIo/RC parameters. An increased ABS/RC ratio indicates that plants grown in P-free solution and plants inoculated with *Codinaeopsis* sp. (328EF), *H. insecticola* (33EF), Biomaphos®, and *E. keratinophilum* (SC4) showed a higher density of absorbed photons per reaction center than PSII, possibly because of the inactivation of some reaction centers or increased antenna size to compensate for energy loss as heat (Schöttler and Tóth, 2014; Urban et al., 2017). This observation suggests competition between the plants and the tested strains, which negatively affects performance (Pi_Abs). Some studies have shown that symbiotic microorganisms can become opportunistic pathogens under excess water conditions, such as in hydroponic systems (Aung et al., 2018). Thus, plants reduce the number of active reaction centers, which is considered a mechanism used by leaves under stress against photo-oxidative damage and excess light energy absorption (Kalaji et al., 2014, 2018). Image fluorescence showed decreased photochemical performance in plants inoculated with *E. keratinophilum* (SC4), *Codinaeopsis* sp. (328EF), and *H. insecticola* (33EF), at physiological stress conditions. Previous studies have described species of these genera as producers of secondary metabolites and antagonists to phytopathogens (e.g., Breinholt et al., 1997; Giridharan et al., 2014; Braga et al., 2018; Puri et al., 2018; Zhu et al., 2021); however, when we forced the interaction of these species with *G. max* plants, biotic stress responses were observed in the plants.

The phytopathogenic opportunism of *Codinaeopsis* sp. (328EF), when interacting with *G. max* roots, was also confirmed by the high sporulation observed *via* electron microscopy during colonization by this strain. Phytopathogenic fungi often sporulate in the presence of host tissues (Su et al., 2012), which is a mechanism used to stimulate the *in vitro* sporulation of this class of fungi (e.g., Crous et al., 2006; Li et al., 2007; Liu et al., 2010). The biotin present in plant tissues may change the synthesis of wall polysaccharides and oleic acid, triggering the selective expression of genes involved in sporulation (Su et al., 2012).

Plants inoculated with *P. alvei* (PA12) and *L. fusiformis* (PA26) presented low ABS/RC and DIo/RC values, which is compatible with non-inoculated plants and indicates the absence of biotic stress promotion by these strains since the potential of these plants to capture photons was not reduced. Plants often try to improve their adaptability under stress by adjusting energy distribution. Therefore, higher DIo/RC values indicate a shift from photochemically active centers to photochemically inactive PSII centers (Malnoë, 2018; Guidi et al., 2019). Likewise, the high Pi_Abs values observed in plants treated with *P. alvei* (PA12) and *L. fusiformis* (PA26) indicate high vitality, which, in general, is due to the significantly increased density of chlorophyll reaction centers. The Pi_Abs value reflects the functionality of both photosystems I and II and represents quantitative information about the current state of plant performance under stress conditions (Strasser et al., 2004). These high values reflected positive *P. alvei* (PA12) and *L. fusiformis* (PA26) inoculation effects on Phi_Po, indicating improved photosynthetic functionality (Ivanov et al., 2017; Dalal and Tripathy, 2018).

### The Hydroponic System Proved the Positive Effect of Microbial Inoculation on P Absorption and Photosynthetic Performance in *G. max*

Plants grown without the inoculation of microorganisms presented tissue P contents compatible with those observed in plants grown in P-free solution, which negatively affected pigment synthesis, photosynthetic performance (Pi_Abs), and pigment development in RVE. Rhizobacteria or growth-promoting fungi can induce metabolic processes that improve photosynthetic and developmental yields (e.g., De Andrade et al., 2019; Bakhshandeh et al., 2020; Kartik et al., 2020; Moretti et al., 2020; Jabborova et al., 2021). Some studies have shown that inoculation can be important under stressful conditions to improve the resilience, development, and productivity of different plant species (Bruno et al., 2020; Forouzi et al., 2020; Prittesh et al., 2020; e.g., Batool et al., 2020).

Component analysis showed that non-inoculated plants were not related to negative fluorescence factors, nor positive developmental and physiological performance factors. Plants under P-free solution and inoculated with microorganisms *Codinaeopsis* sp. (328EF), *H. insecticola* (33EF), Biomaphos®, and *E. keratinophilum* (SC4) were strongly correlated with DIo/RC, ABS/RC, TRo/RC, and Ci parameters, which are negatively related to positive biometric and physiological patterns, reinforcing the hypothesis that these patterns constitute biotic stress responses in *G. max*.

## Conclusion

Calcium phosphate solubilization results obtained using a hydroponic system were inconsistent with those observed in solid and liquid media; however, tests in liquid medium demonstrated the poor performance of *Codinaeopsis* sp. (328EF) and *H. insecticola* (33EF) in reducing the pH and levels of solubilizing phosphates, corroborating the effects of biotic stress observed in *G. max* plants inoculated with these strains. On the other hand, the hydroponic system allowed the characterization of *P. alvei* (PA12), which was also efficient in solubilizing CaHPO_4_ in a liquid medium, and *L. fusiformis* (PA26) was the most effective in CaHPO_4_ solubilization because of its better effects on P absorption, growth promotion, and physiological performance observed in plants inoculated with these bacteria. Therefore, the use of a hydroponic system indicated strains with higher potential than Biomaphos® for growth promotion. These results demonstrated the effectiveness of this approach in confirming the functional traits of strains previously selected as solubilizers, as it allowed the evaluation of tissue nutritional content patterns, growth patterns, photosynthetic performance, anatomical patterns, and even the detection of biotic stress responses to inoculation, the latter making the use of strains unfeasible.

## Data Availability Statement

The original contributions presented in the study are included in the article/Supplementary Material, further inquiries can be directed to the corresponding author/s.

## Author Contributions

MR: methodology, investigation, and writing—original draft. LB: supervision, resources, and writing—review and editing. AJ: methodology and investigation. FG and MM: writing—review and editing. LV: conceptualization, investigation, resources, and writing—review and editing. All authors contributed to the article and approved the submitted version.

## Conflict of Interest

The authors declare that the research was conducted in the absence of any commercial or financial relationships that could be construed as a potential conflict of interest.

## Publisher's Note

All claims expressed in this article are solely those of the authors and do not necessarily represent those of their affiliated organizations, or those of the publisher, the editors and the reviewers. Any product that may be evaluated in this article, or claim that may be made by its manufacturer, is not guaranteed or endorsed by the publisher.
