# Supplementary material for: Efficiency of the Hydroponic System as an Approach to Confirm the Solubilization of CaHPO4 by Microbial Strains Using Glycine max as a Model
Source: Front Plant Sci. 2021 Oct 29;12:759463. doi: 10.3389/fpls.2021.759463 (PMC8589188; doi:10.3389/fpls.2021.759463)
Supplement: Supplementary file 1 [file Data_Sheet_1.docx]

Supplementary Material

## Supplementary Figures

**
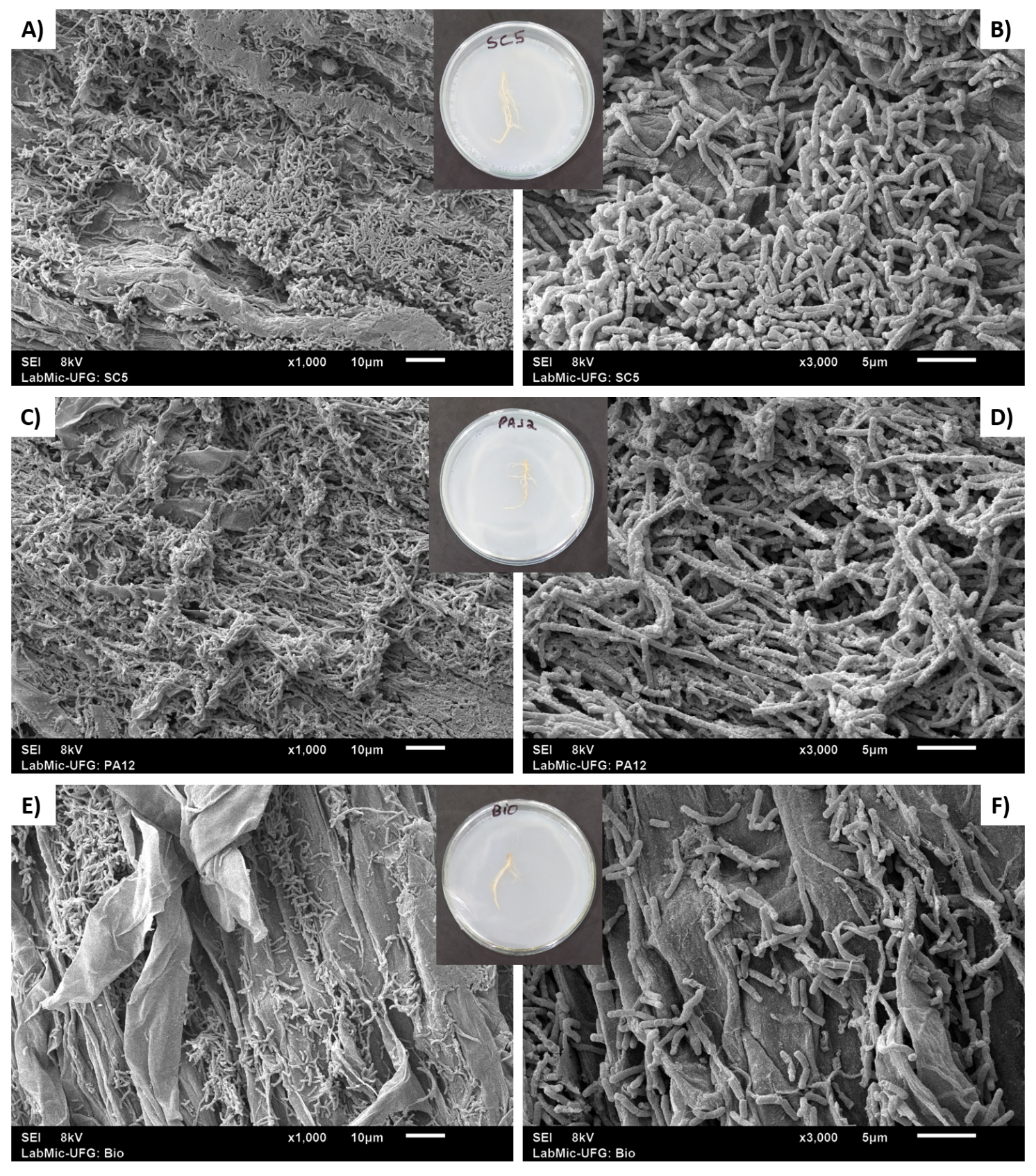
**

**Supplementary Figure 1.** Microscopic aspects of root colonization in soybean plants (*Glycine max)* treated with the bacterial strains SC5 = *Bacillus cereus* (A and B) and PA12 = *Paenibacillus alvei* (C and D), and Biomaphos^®^ (E and F).

**
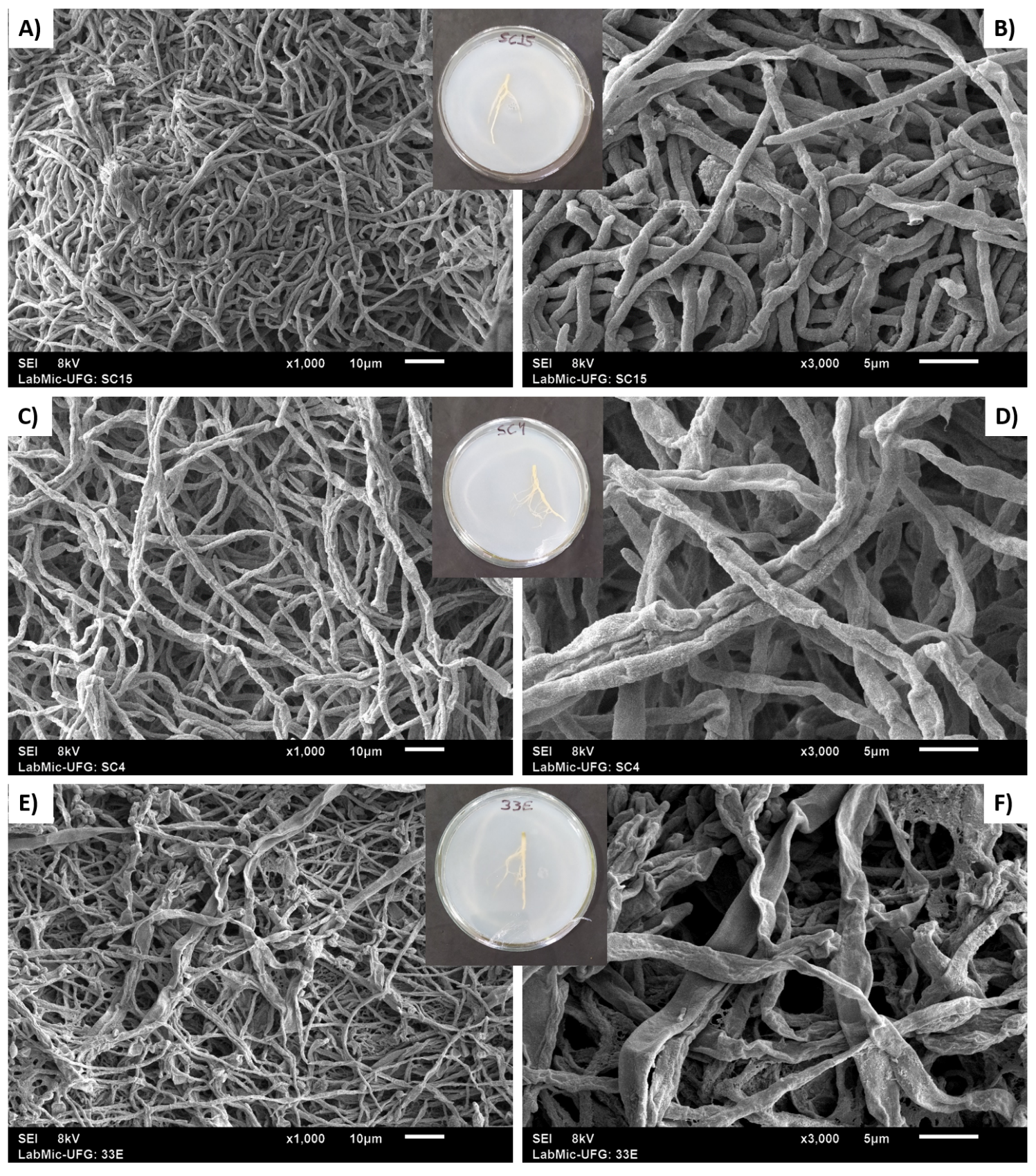
**

**Supplementary Figure 2.** Microscopic aspects of root colonization in soybean plants (*Glycine max)* treated with three fungal strains. SC15 = *Penicillium sheari* (A and B), SC4 = *Epicoccum keratinophilum* (C and D) and 33EF = *Hamigera insecticola* (E and F).


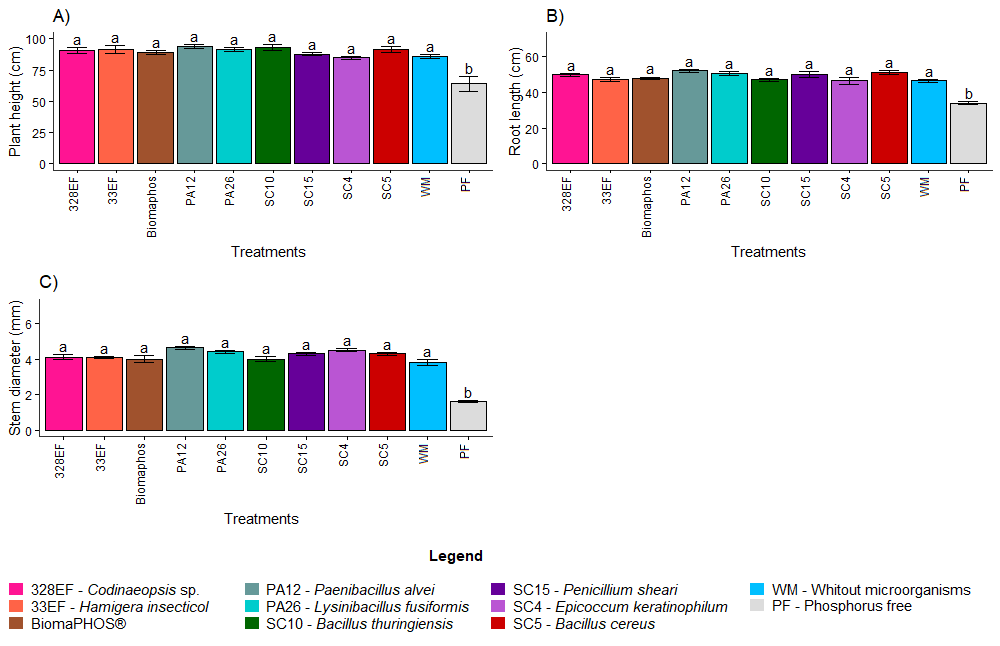


**Supplementary Figure 3.** Plant height (A), root length (B) and stem diameter (C) in soybean plants (*Glycine max)* grown in nutrient solution providing calcium phosphate (CaHPO_4_) as a phosphate source and inoculated with four bacterial strains and four fungal strains. Means followed by the same letter do not differ from one another according to the Scott-Knott test at 5% probability.

**
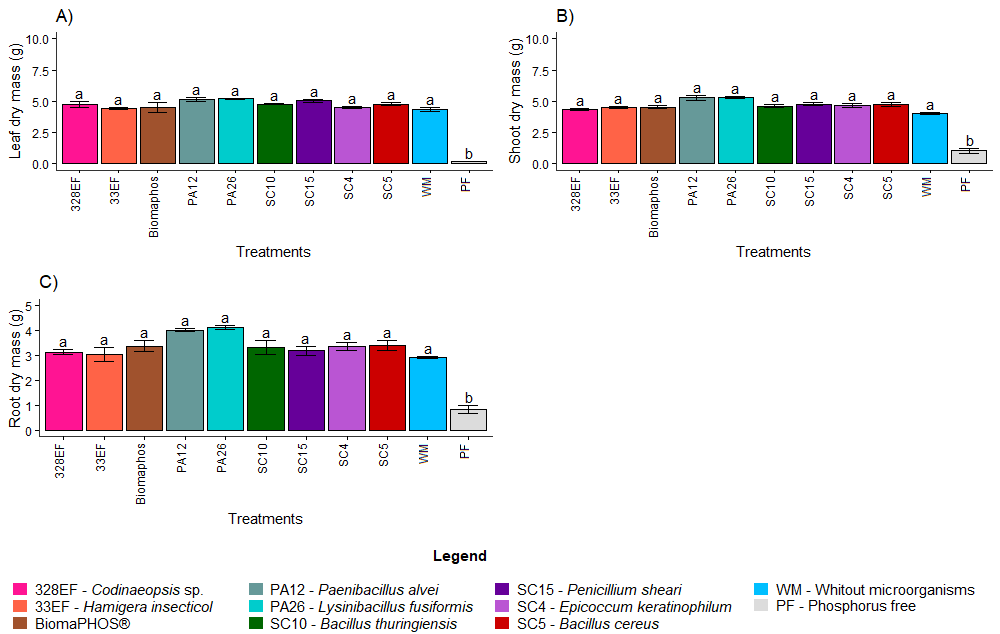
**

**Supplementary Figure 4.** Leaf dry mass (A), shoot dry mass (B) and root dry mass (C) in soybean plants (*Glycine max*) grown in nutrient solution providing calcium phosphate (CaHPO_4_) as a phosphate source and inoculated with four bacterial strains and four fungal strains. Means followed by the same letter do not differ from one another by the Scott-Knott test at 5% probability.

**
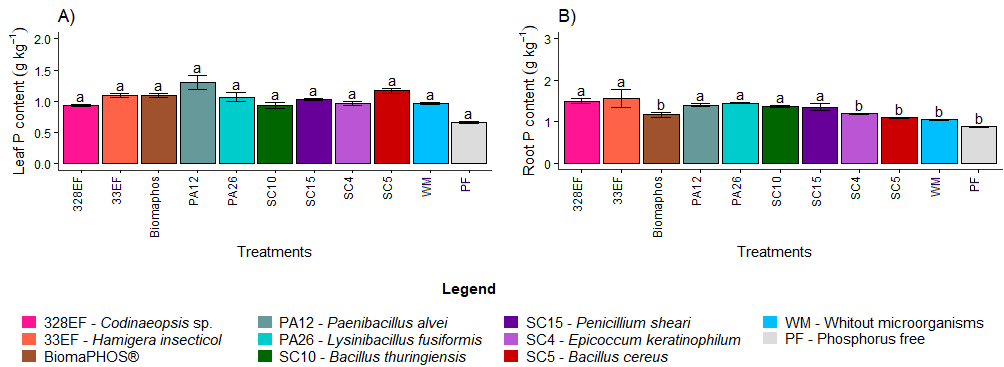
**

**Supplementary Figure 5.** P tissue content in the leaves (A) and roots (B) in soybean plants (*Glycine max*) grown in nutrient solution providing calcium phosphate (CaHPO_4_) as a phosphate source and inoculated with four bacterial strains and four fungal strains. Means followed by the same letter do not differ from one another according to the Scott-Knott test at 5% probability.


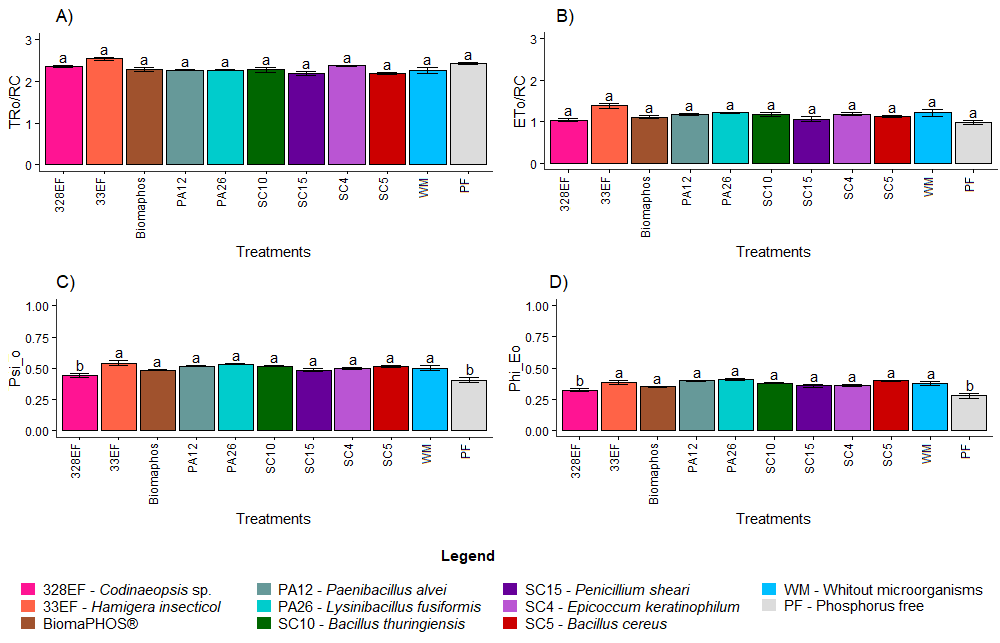


**Supplementary Figure 6.** Energy flow captured by RC at t = 0 (TRo/RC) (A), electron transport flow by RC at t = 0 (ETo/RC) (B), probability of an exciton moving an electron through the electron transport chain after quinone (Qa) (PSI_O) (C) and electron transport quantum yield (PHI_Eo) (D) in soybean plants (*Glycine max)* grown in nutrient solution providing calcium phosphate (CaHPO_4_) as a phosphate source and inoculated with four bacterial strains and four fungal strains. Means followed by the same letter do not differ from one another according to the Scott-Knott test at 5% probability.
